# Clostridium difficile infection in medical wards – a mathematical model

**DOI:** 10.1186/2047-2994-4-S1-O34

**Published:** 2015-06-16

**Authors:** V Schechner, Y Carmeli, M Leshno

**Affiliations:** 1 Tel Aviv Sourasky Medical Center, Tel Aviv, Israel; 2 Tel Aviv University, Tel Aviv, Israel

## Introduction

*Clostridium difficile* infection (CDI) is a common and potentially fatal healthcare-associated infection. Better isolation of CDI patients may prevent transmission and reduce CDI cases.

## Objectives

To determine 1) the dynamics of CDI, and 2) the association between CDI transmission and prevalence of CDI, among hospitalized patients in internal medicine wards.

## Methods

Daily reports were created retrospectively to obtain the number of hospitalized CDI patients per day in the 350 beds internal medicine department for 3 years (March 2010 to February 2013); data were evaluated for stationarity and autocorrelation. A mathematical model of CDI transmission was constructed based on hospital data. The model consisted of three compartments: susceptible patients, asymptomatic carriers and CDI patients. We used the model results to assess the transmission rate from infected patients under different infection control scenarios.

## Results

The number of CDI patients in the internal medicine department is stationary; the autocorrelation function of this parameter is high (i.e., 90%) for a lag time of 1 day and it decreases gradually thereafter (i.e., 56% for a lag of 7 days, and 44% for a lag of 14 days). The average number of CDI cases increases exponentially as the transmission rate increases; a major reduction in the number of CDI patients (from 18.0 to 8.5 patients per 350 beds) can be achieved by modestly lowering the transmission rate through contact isolation precautions, but further reduction in the average number of infected cases requires more substantial changes in the transmission rate. The basic reproduction rate during the study period, as derived from the model results, was calculated as 1.09 indicating endemicity

## Conclusion

Hospitals should be encouraged to improve patient isolation in order to reduce CDI cases.

## Disclosure of interest

None declared.

